# HRSA’s evidence-based tele-emergency network grant program: Multi-site prospective cohort analysis across six rural emergency department telemedicine networks

**DOI:** 10.1371/journal.pone.0243211

**Published:** 2021-01-12

**Authors:** Sarah Heppner, Nicholas M. Mohr, Knute D. Carter, Fred Ullrich, Kimberly A. S. Merchant, Marcia M. Ward

**Affiliations:** 1 Federal Office of Rural Health Policy, Health Resources and Services Administration, U.S. Department of Health and Human Services, Rockville, Maryland, United States of America; 2 Department of Emergency Medicine, University of Iowa Carver College of Medicine, Iowa City, Iowa, United States of America; 3 Department of Anesthesia Critical Care, University of Iowa Carver College of Medicine, Iowa City, Iowa, United States of America; 4 Department of Epidemiology, University of Iowa College of Public Health, Iowa City, Iowa, United States of America; 5 Department of Biostatistics, University of Iowa College of Public Health, Iowa City, Iowa, United States of America; 6 Department of Health Management and Policy, University of Iowa College of Public Health, Iowa City, Iowa, United States of America; University of Oklahoma Health Sciences Center, UNITED STATES

## Abstract

**Background:**

The Health Resources and Services Administration (HRSA), Federal Office of Rural Health Policy (FORHP) funded the Evidence-Based Tele-Emergency Network Grant Program (EB TNGP) to serve the dual purpose of providing telehealth services in rural emergency departments (teleED) and systematically collecting data to inform the telehealth evidence base. This provided a unique opportunity to examine trends across multiple teleED networks and examine heterogeneity in processes and outcomes.

**Method and findings:**

Six health systems received funding from HRSA under the EB TNGP to implement teleED services and they did so to 65 hospitals (91% rural) in 11 states. Three of the grantees provided teleED services to a general patient population while the remaining three grantees provided teleED services to specialized patient populations (i.e., stroke, behavioral health, critically ill children). Over a 26-month period (November 1, 2015 –December 31, 2017), each grantee submitted patient-level data for all their teleED encounters on a uniform set of measures to the data coordinating center. The six grantees reported a total of 4,324 teleED visits and 99.86% were technically successful. The teleED patients were predominantly adult, White, not Latinx, and covered by Medicare or private insurance. Across grantees, 7% of teleED patients needed resuscitation services, 58% were rated as emergent, and 30% were rated as urgent. Across grantees, 44.2% of teleED patients were transferred to another inpatient facility, 26.0% had a routine discharge, and 24.5% were admitted to the local inpatient facility. For the three grantees who served a general patient population, the most frequent presenting complaints for which teleED was activated were chest pain (25.7%), injury or trauma (17.1%), stroke symptoms (9.9%), mental/behavioral health (9.8%), and cardiac arrest (9.5%). The teleED consultation began before the local clinician exam in 37.8% of patients for the grantees who served a general patient population, but in only 1.9% of patients for the grantees who provided specialized services.

**Conclusions:**

Grantees used teleED services for a representative rural population with urgent or emergent symptoms largely resulting in transfer to a distant hospital or inpatient admission locally. TeleED was often available as the first point of contact before a local provider examination. This finding points to the important role of teleED in improving access for rural ED patients.

## Introduction

Telehealth is increasingly being used to improve access to specialized emergency care for the approximately 60 million rural residents in the United States [1, 2]. Emergency departments (EDs) are a key source of care for rural communities and often face challenges with providing specialty care, such as trauma and behavioral health [3, 4]. The low volume seen in most rural EDs and the unpredictable nature of emergency care can make it difficult for rural hospitals to provide specialty services. For example, in many rural communities the majority of physicians covering the ED are family physicians rather than emergency specialists [5, 6]. Previous studies have found that telehealth in rural EDs can lead to improved patient care and support for rural providers [7–12].

In September 2014 the Federal Office of Rural Health Policy (FORHP), located in the U.S. Department of Health and Human Services’ Health Resources and Services Administration (HRSA), launched the Evidence-Based Tele-Emergency Network Grant Program (EB TNGP). This innovative new program allowed HRSA and its stakeholders to use grant funding to serve the dual purpose of delivering ED consultation services via telehealth to rural hospitals without emergency medicine specialists and systematically collecting data to inform the evidence base assessing the effectiveness of tele-emergency (teleED) care for rural populations. Compiling a cohort of otherwise unconnected provider-to-provider ED-based telehealth networks into a unified analysis offered a unique opportunity to generate a significant sample of rural telehealth encounters.

This program, administered by the Office for the Advancement of Telehealth, which is located in HRSA/FORHP, was developed in response to recommendations from the National Advisory Committee on Rural Health and Human Services. The committee advised HRSA to inform the evidence base by targeting its grant funds at specific areas of clinical telehealth services and analyzing the impact of those services. These recommendations were informed in part by a 2012 Institute of Medicine workshop entitled “The Role of Telehealth in an Evolving Health Care Environment” which was convened with financial support from a HRSA contract [13]. A published summary of this meeting noted the challenges in generating statistically significant results in small rural areas that demonstrate the impact of telehealth and inform health care policy [13]. These organizations stressed the need to increase the evidence base for telehealth services with a specific focus on rural populations. HRSA is in a unique position to utilize its grant-making authority to simultaneously increase access to care in rural areas through the use of telehealth and support rigorous analysis to inform the evidence base.

The purpose of this study was to describe the hospital and patient characteristics of those providing and receiving teleED services, including the indications, success, and outcomes of teleED encounters, and to compare the use of teleED between general and specialized teleED services. Our hypotheses were that teleED would be used for broad indications in general teleED networks and very narrow indications in specialized targeted networks, that it would be most commonly used with the sickest patients, and that it would be technically successful in these established networks.

## Methods

### Study design

This study was a prospective cohort study of patients treated with teleED in any of the six grantee rural teleED networks. For the purposes of this study, teleED was defined as an immediate, synchronous, interactive audio/video connection between an ED originating site (spoke) and a distant site where a specialist is located (hub). Participating networks collected a defined uniform set of measures and submitted those data to the data coordinating center for pooling and analysis.

### Participating networks

Six health systems received grant funding from HRSA’s FORHP under the EB TNGP to implement teleED services. The six grantees were: Avera Health, Saint Vincent Healthcare, Union Hospital, University of California–Davis, University of Kentucky, and University of Virginia. The six grantees provided teleED services to 65 hospitals in 11 states–California, Indiana, Iowa, Kansas, Kentucky, Minnesota, Montana, Nebraska, North Dakota, South Dakota, and Virginia. All six organizations had at least ten years of experience in offering telehealth services to their regions and were continually improving and expanding telehealth services.

Grantees provided teleED services customized to the needs and resources in their areas for specific project goals. In particular, three of the grantees (Avera Health, St. Vincent Healthcare, University of Kentucky) provided teleED services to a general patient population (i.e., any patient seen in the ED was eligible for teleED) while the remaining three grantees provided teleED services to specialized patient populations (behavioral health and neurology at Union Hospital; pediatric emergency/critical care at University of California–Davis; stroke care at University of Virginia). At the three general teleED networks, the hub sites were staffed by board-certified emergency physicians. At the three specialized teleED networks, the hub sites were staffed by specialty physicians (e.g., psychiatrists, pediatric critical care physicians, neurologists and vascular physicians). Five of the six hub sites used on-call physicians, while the sixth hub had dedicated staff on site.

### Measure development

HRSA awarded a contract in Fall 2014 to Mathematica Policy Research to identify a set of evidence-based measures that would be appropriate for use in teleED studies. University of Iowa investigators were subcontracted for the project and completed a systematic review of the teleED literature and compiled a library of existing measures for consideration [12]. A set of measures that could be applied to all teleED encounters and a set of measures that could be applied to teleED encounters for patients with specific conditions were identified. Mathematica Policy Research worked with several EB TNGP grantees to pilot test the measures, then developed and refined a data collection tool (Tele-Emergency Performance Assessment Reporting Tool, T-PART) [14]. University of Iowa investigators in the Rural Telehealth Research Center (RTRC), funded under a HRSA cooperative agreement, continued refinement of the standardized data elements and T-PART before serving as the data coordinating and analysis center for the EB TNGP [15].

### Data collection

From November 1, 2015 to December 31, 2017, each grantee submitted patient-level data for all their teleED encounters to RTRC [15]. Inclusion criteria encompassed all patients seen by each network through their teleED program. Data on 49 data elements were submitted on a predetermined schedule by each grantee using the Microsoft Excel (Microsoft Corporation, Redmond, WA)-based T-PART tool [15]. RTRC provided the grantees with an extensive data dictionary which included definitions, allowable data values, and guidelines for data abstraction [15]. RTRC used data checking algorithms and worked iteratively with grantees to address missing and out-of-range data submissions. Data elements reported in these analyses include patient demographics (age, sex, race, and ethnicity reported using U.S. Census definitions), insurance coverage, technical success, weekday and time of ED visit and teleED consult, severity of illness, and ED disposition. Severity of illness was reported by grantees using the Emergency Severity Index, a validated measure of patient acuity designed to rate the urgency of needed care and predict ED resource consumption [16–18].

### Additional data sources

Spoke hospital characteristics were abstracted from the 2016 American Hospital Association annual survey [19]. Rural-Urban Commuting Area codes were used to define spoke hospitals as located in metro or rural locations [20].

### Analysis

The primary analysis is descriptive, reporting counts and proportions. Categorical variables were compared with chi-squared tests or Fisher’s exact test, as appropriate. Continuous variables were compared with t-tests and Wilcoxon rank sum tests, as appropriate. All statistical tests were conducted as two-tailed tests with significance defined as p<0.05. The sample size of this observational study was selected to be the entire population of interest based on the 26-month enrollment period. Because all study procedures occurred in the initial visit, no cases were lost to follow-up. Missing data were not imputed, and summary data are reported of complete data on each data element. The study protocol was approved by the Institutional Review Board at the RTRC (University of Iowa Institutional Review Board) and each of the grantee organizations (Avera Institutional Review Board, Indiana State University Institutional Review Board, Rector and Visitors of the University of Virginia Intuitional Review Board, St. Vincent Institutional Review Board, University of California, Davis Institutional Review Board Administration, and University of Kentucky Institutional Review Board) and data use agreements defined data sharing arrangements between all entities. No personal health information was transmitted and all data were anonymized prior to transfer. The findings are reported in accordance with the Strengthening The Reporting of Observational Studies in Epidemiology (STROBE) guidelines [21].

## Results

Over the 26-month data collection period, the six grantees reported a total of 4,324 ED visits for which teleED was used.

### Hospital characteristics

The 65 spoke hospitals participating in the teleED networks were predominantly in rural locations (90.8%). Spoke hospitals included 44 (67.7%) Critical Access Hospitals and 21 (32.3%) Prospective Payment System) hospitals. General teleED networks were more likely to include Critical Access Hospitals (81.1%) than Prospective Payment System hospitals (18.9%) while the specialized teleED networks had an even split (50% Critical Access Hospitals, 50% Prospective Payment System hospitals). The median ED annual volume was 8,427 visits across spoke hospitals. General teleED network hospitals had an annual median of 2,437 ED visits compared to 13,760 for specialized teleED network hospitals, consistent with their respective Critical Access Hospital/Prospective Payment System hospital status.

### Patient characteristics

The demographics of the EB TNGP telehealth patient population are shown in Table 1 for the total group and for general teleED networks vs. specialized teleED networks. Because of the condition-specific criteria for the specialized teleED services, the demographics of the teleED patients differed significantly between the two groups. Overall, the teleED patients were predominantly adult, White, not Latinx, and covered by Medicare or private insurance.

**Table 1:** Demographics of the EB TNGP TeleED patient population.

	Total teleED sample		Telehealth cases at hospitals participating in grantee networks with general teleED services		Telehealth cases at hospitals participating in grantee networks with specialized teleED services		p value
	N	Percent	N	Percent	N	Percent	
**Patient age**							
<1	99	2.3%	32	1.0%	67	5.7%	<0.0001
1–14	370	8.6%	205	6.5%	165	14.1%	
15–17	108	2.5%	107	3.4%	1	0.1%	
18–24	303	7.0%	212	6.7%	91	7.8%	
25–44	657	15.2%	481	15.3%	176	15.0%	
45–64	1206	27.9%	908	28.8%	298	25.5%	
65–74	722	16.7%	558	17.7%	164	14.0%	
75 or older	858	19.8%	650	20.6%	208	17.8%	
Missing	1	0.02%	1	0.03%	0	0.0%	
**Patient sex**							
Female	1,958	45.3%	1379	43.7%	579	49.5%	0.0007
Male	2,366	54.7%	1775	56.3%	591	50.5%	
**Patient race**							
American Indian/Alaska Native	240	5.6%	239	7.6%	1	0.1%	<0.0001
Asian	15	0.4%	7	0.2%	8	0.7%	
Black/African-American	108	2.5%	24	0.8%	84	7.2%	
Native Hawaiian/Other Pacific Islander	2	0.1%	1	0.0%	1	0.1%	
White	3,555	82.2%	2597	82.3%	958	81.9%	
Multi-racial	6	0.1%	0	0.0%	6	0.5%	
Missing	398	9.2%	286	9.1%	112	9.6%	
**Patient ethnicity**							
Hispanic/Latino	149	3.5%	94	3.0%	55	4.7%	0.0004
Not Hispanic/Latino	3,266	75.5%	2385	75.6%	881	75.3%	
Unknown	712	16.5%	478	15.2%	234	20.0%	
Missing	197	4.6%	197	6.3%	0	0.0%	
**Insurance coverage (Primary payer)**							
Dual Medicare/Medicaid	98	2.3%	15	0.5%	83	7.1%	<0.0001
Medicaid only	772	17.9%	425	13.5%	347	29.7%	
Medicare only	1646	38.1%	1303	41.3%	343	29.3%	
Private Insurance	1308	30.3%	1022	32.4%	286	24.4%	
Self-pay/uninsured	326	7.5%	240	7.6%	86	7.4%	
Indian Health Service	64	1.5%	64	2.0%	0	0.0%	
Veterans Administration	13	0.3%	6	0.2%	7	0.6%	
Corrections Health	6	0.1%	6	0.2%	0	0.0%	
Other	70	1.6%	62	2.0%	8	0.7%	
Missing	21	0.5%	11	0.4%	10	0.9%	

Percentages may not sum to 100% due to rounding.

### TeleED presenting complaint

**Fig 1:**
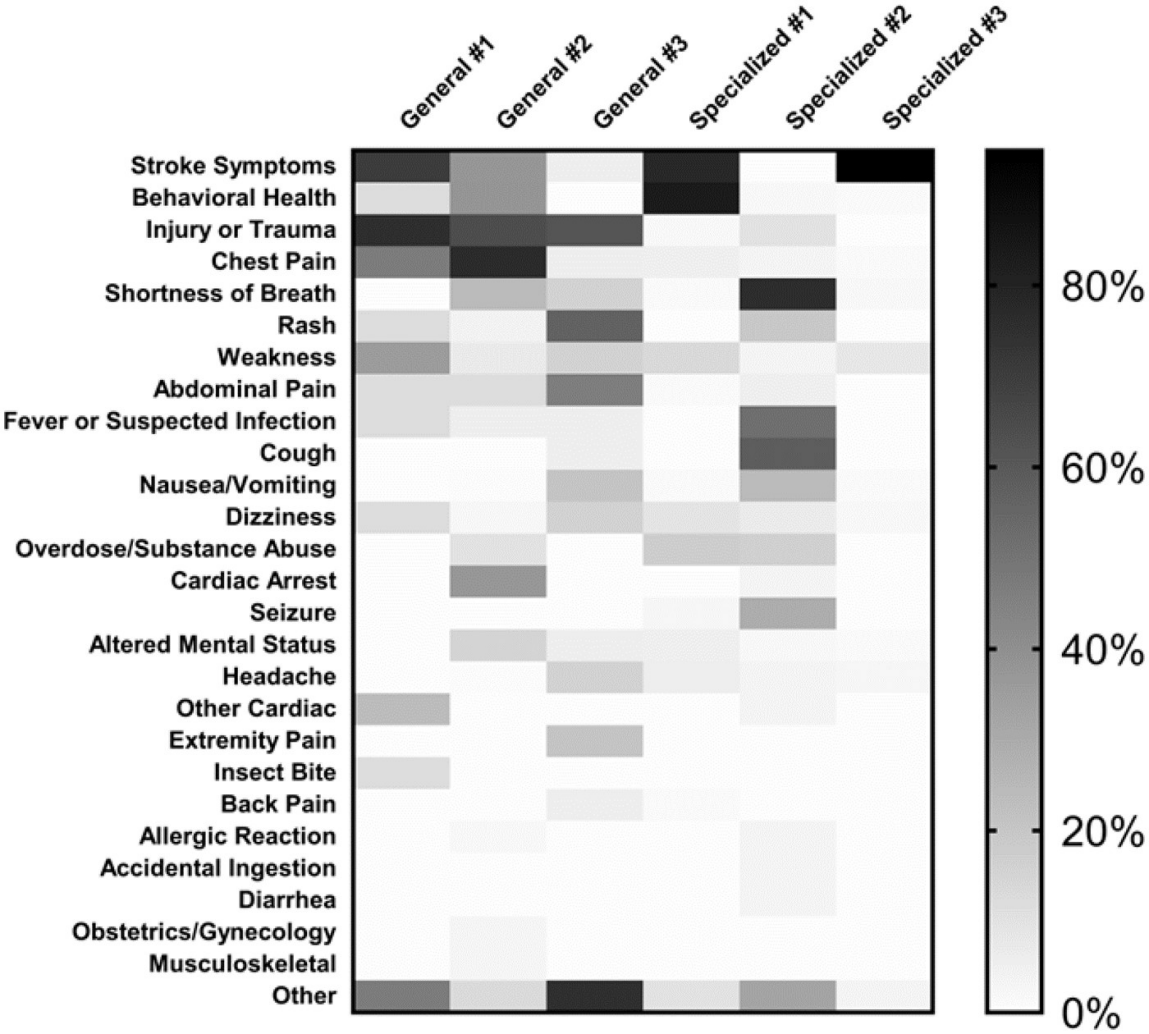
Distribution of chief complaint by network. Networks are divided into General teleED Networks or Specialized teleED Networks. Chief complaints were assigned at the beginning of a teleED encounter. The shade of each box indicates the proportion of cases treated within the teleED network that fall within a chief complaint category. The legend to the right shows the shade associated with each proportion.

Presenting complaints for the patients where teleED was activated differed for the three grantees who provided general teleED services compared to the three grantees who provided specialized teleED services (p<.0001). For the grantees who provided teleED services to a general patient population, the most frequent presenting complaints for which teleED was activated were chest pain (25.7%), injury or trauma (17.1%), stroke symptoms (9.9%), mental/behavioral health (9.8%), and cardiac arrest (9.5%). In contrast, grantees who provided specialized teleED services had chief complaints distributed to match the services on which the network focused. In particular, two of the grantees provided teleED specialized stroke services and one of the grantees provided teleED specialized behavioral health services, so the most frequent presenting complaints for these grantees aligned with these services. The distribution of chief complaints (by network) are summarized in Fig 1.

### TeleED processes

Successful administration was defined as encounters where voice and video quality were sufficient to complete the consultation. Unsuccessful administration was defined as encounters where the voice and/or video quality (e.g. unreachable network, poor image quality, poor ECG signal quality) were not sufficient to complete the consultation. Only 6 of the 4,324 teleED encounters were judged to be unsuccessful, resulting in 99.86% of teleED encounters technically successful.

The distribution of teleED encounters was uniform across day of week, with the highest utilization on Thursdays (15.0%) and the lowest on Wednesdays (13.3%). Among teleED encounters, a higher proportion occurred for patients who arrived at the ED during business hours (7:30 am to 5:30 pm) compared to outside business hours (53.8% and 46.2%, respectively), and the difference was particularly evident for the grantees with general teleED services, as shown in Table 2.

**Table 2:** Time of day of TeleED encounter.

Time of day of teleED encounter	Telehealth cases at hospitals participating in grantee networks with general teleED services		Telehealth cases at hospitals participating in grantee networks with specialized teleED services		p value
	N	Percent	N	Percent	
Business hours (0730–1730)	1732	54.9%	594	50.8%	0.0151
Not business hours (>1730 -<0730)	1422	45.1%	576	49.2%	

### Timing of TeleED consultation

The sequence of consultation differed significantly for the three grantees who provided teleED services to a general patient population compared to the three grantees who provided teleED services to specialized patient populations (Table 3). Grantees providing general teleED services began consultations prior to local provider evaluation much more frequently than those providing specialized teleED care (37.8% vs 1.9%, p<0.0001).

**Table 3:** Timing of TeleED consultation versus local clinician exam.

Timing of TeleED consultation in relationship to when patient was first examined by a local clinician	Telehealth cases at hospitals participating in grantee networks with general teleED services	Telehealth cases at hospitals participating in grantee networks with specialized teleED services	p value
TeleED consultation began > 1 hour before local clinician exam	1.5%	0.0%	<0.0001
TeleED consultation began < = 1 hour before local clinician exam	36.3%	1.9%	
TeleED consultation began at the same time as local clinician exam	8.2%	0.6%	
TeleED consultation began < = 1 hour after local clinician exam	42.3%	49.7%	
TeleED consultation began > 1 hour after local clinician exam	11.9%	47.8%	

Percentages may not sum to 100% due to rounding.

### Severity of illness

Analysis of the Emergency Severity Index (ESI) indicated that across grantees, 7% needed resuscitation services (ESI level 1), 58% of teleED patients were rated as emergent (ESI level 2), and 30% were rated as urgent (ESI level 3). ESI did not differ significantly for the patients seen by grantees delivering services to a general patient population compared to grantees delivering specialized services (p = 0.211). However, the specialized teleED services took longer to activate the teleED consultation across all ESI levels, even those that were most serious (Table 4, p<0.0001). Unfortunately, because ESI was not routinely collected from all rural hospitals, this was the variable with the greatest missingness (e.g., 44% of participating cases from one grantee).

**Table 4:** Emergency severity index of TeleED patients and median time of TeleED consultation in relationship to when patient was first examined by a local clinician. The proportion of cases within each ESI category are different between telehealth cases in General and Specialized teleED networks (p<0.001).

Emergency Severity Index of TeleED Patients	Telehealth cases at hospitals participating in grantee networks with general teleED services		Telehealth cases at hospitals participating in grantee networks with specialized teleED services		p-value (comparing median time to evaluation in each strata of networks)
	Percent	Median Time (IQR)	Percent	Median Time (IQR)	
1-Resuscitation	7.8%	-1.0 min (-6–3)	6.4%	29.0 min (18–44)	<0.0001
2-Emergent	58.4%	2.0 min (-5–20)	58.9%	45.0 min (25–96)	<0.0001
3-Urgent	30.2%	7.0 min (-6–48)	29.8%	81.0 min (36–160)	<0.0001
4-Less urgent	3.0%	11.0 min (4–63)	4.1%	115.0 min (85–210)	<0.0001
5-Non-urgent	0.6%	17.5 min (5–55)	0.9%	54.0 min (26–145)	0.1081

Percentages may not sum to 100% due to rounding.

### TeleED outcomes

The discharge status of all patients receiving teleED services was examined. Over all six grantees, the most common disposition was transfer to another inpatient facility (44.2%), followed by routine discharge (26.0%), and admission to the local inpatient facility (24.5%). As shown in Table 5, discharge status differed for grantees delivering services to a general patient population compared to grantees delivering specialized services, with the specialized teleED services transferring and admitting more patients than the general teleED services (p<.0001).

**Table 5:** Discharge status of TeleED patients. The patient disposition is different in General teleED networks vs Specialized teleED networks (p<0.001).

Discharge Status of TeleED Patients	Telehealth cases at hospitals participating in grantee networks with general teleED services		Telehealth cases at hospitals participating in grantee networks with specialized teleED services		p value
	N	Percent	N	Percent	
Transferred to another inpatient facility	1383	43.9%	551	47.1%	<0.0001
Admitted to local inpatient facility	730	23.2%	329	28.1%	
Routine discharge	871	27.6%	253	21.6%	
Died in ED	140	4.4%	2	0.2%	
Other (left against medical advice, corrections, observation)	30	0.9%	35	3.0%	

Percentages may not sum to 100% due to rounding.

## Discussion

TeleED services are one tool available to address challenges with access to specialty care in rural EDs. This study describes hospital and patient characteristics of those served by specialized and generalized tele-ED programs, across a cohort of established networks. Our findings show that teleED is used for a variety of presenting urgent and emergent conditions largely resulting in transfer to a distant hospital or inpatient admission locally and it provided timely access to care that would otherwise be unavailable. These findings also show that in these established networks, teleED was nearly always technically successful, which aligns with prior research showing that technical success is not a barrier to teleED use [12, 22]. This study illustrates how the structure and goals of networks affect the application and function for their participating spoke hospitals and patients.

Access to care is one of the primary drivers for telehealth in rural areas observed in this study. Interestingly, 37.8% of generalized ED provider encounters began prior to evaluation by a local health care provider. This telehealth-first strategy occurs primarily in hospitals without a dedicated ED provider. In many of these facilities, the provider responsible for covering the ED may be in clinic, seeing patients elsewhere in the hospital, or on call from home. A 2013 memorandum from the Centers for Medicare & Medicaid Services (CMS) clarified that Critical Access Hospitals could use a physician at a telehealth hub to fulfill the requirements under their conditions of participation and the Emergency Medical Treatment and Active Labor Act (EMTALA) related to physician coverage for the ED [23]. In these low-volume EDs, telehealth can provide immediate access to a provider who can perform an evaluation, order tests, and arrange transportation for transferring patients, if appropriate. Tele-ED, paired with the policy clarification from CMS, increases rural hospitals’ options to address physician shortages and challenges with ED coverage, ultimately improving access to care for rural communities [24]. This focus on access to care reflects one of the priorities of a multi-stakeholder panel convened by the National Quality Forum, with funding from a contract with CMS to propose a telehealth measurement framework [25].

One interesting finding is the breadth of conditions for which teleED was consulted. Prior reports have described single network experiences [26, 27] or condition-specific telehealth interventions [28–30]. While many networks have developed telehealth infrastructure for cardiac emergencies, trauma, and stroke, that infrastructure is often used for other clinical scenarios where expert consultation may be valued. This was particularly apparent in the specialty-specific networks, where 5% of the encounters reported conditions outside the focus area of the network. Our study was unable to measure the impact of these off-specialty consultations, but local providers may have viewed the telehealth infrastructure as an option for patients who otherwise were difficult to manage. Even in the generalized networks, only 72.1% of encounters were for the top five chief complaints, suggesting that infrequent and unusual presentations may be over-represented in patients for whom telehealth is consulted.

The largest prior report of teleED service implementation came from the University of Mississippi TelEmergency network (n = 26,697 from 1 network) [27]. In that service, very strict consultation criteria were applied because all participating spoke hospital EDs were staffed by nurse practitioners overseen by faculty at the University of Mississippi ED. Over a 3-year period, over 40% of all ED patients in 10 rural centers used teleED, and most of those patients (65%) were discharged to home. The intervention was paired with a quality improvement initiative, and the patients in their cohort (compared with our study) were much younger with a much lower proportion of chest pain, trauma, and stroke. These markedly different distributions of users reflect the heterogeneity in telehealth adoption, and highlight the importance of cross-network data set analysis.

One challenge in telehealth research is predicting the long-term impact of telehealth programs at scale. Pilot projects, single-center analyses, and demonstration programs may over-estimate the impact of novel care delivery mechanisms. With tele-stroke programs, for example, the earliest studies showed the greatest benefits, although consistent benefit was shown even in later studies [31]. Very few analyses of telehealth interventions have pooled data from multiple networks, and a systematic review of teleED implementation papers did not identify any multi-network studies [12]. The goal of the EB TNGP program was to pool heterogeneous networks that have expanded to scale to better estimate how teleED might reshape rural health care if it were expanded beyond the networks that pioneered its use [32–35]. Observing the variation between networks in their application and use increases confidence in how teleED might perform at scale, and it allows for better prediction of the impact of systematic telehealth expansion into new health systems.

## Limitations

This study has several limitations. In order to maximize feasibility and participation, the number of data elements collected for the T-PART instrument was limited. Second, this data set does not collect long-term clinical outcomes. By restricting outcomes to the ED, we have no significant loss to follow-up based on our data set. Third, comparing the racial distribution in this sample to the US Census rural population in the 11 states represented, the American Indian/Native Alaska percentage was about twice that expected and the Black/African American was about a third that expected.

## Conclusion

TeleED services are used across a variety of clinical conditions in rural EDs, and the indication for use, the timeliness of telehealth activation, and the ultimate ED disposition of patients varies based on the structure, design, and purpose of the provider-to-provider network being studied. HRSA’s Evidence-Based Tele-Emergency Network Grant Program has assembled a large dataset using uniform measures to enable cross-network collaborative research, and unifying projects like this will continue to enable objective evaluation of novel care delivery systems. Future work should continue to evaluate the impact of teleED on costs of care, clinical outcomes, and long-term recovery from acute illness, and strong collaborative projects such as this can enable these comparative effectiveness studies.

## Supporting information

S1 Checklist STROBE statement—Checklist of items that should be included in reports of observational studies. (DOCX)
